# Moderate endurance training (marathon-training) – effects on immunologic and metabolic parameters in HIV-infected patients: the 42 KM cologne project

**DOI:** 10.1186/s12879-017-2651-y

**Published:** 2017-08-08

**Authors:** Stefan Schlabe, Martin Vogel, Christoph Boesecke, Carolynne Schwarze-Zander, Jürgen K. Rockstroh, Christian Körner, Klara Brixius, Jan-Christian Wasmuth

**Affiliations:** 1 Department of Internal Medicine I, University of Bonn, Sigmund-Freud-Str. 25, 53127 Bonn, Germany; 2 Department of Molecular and Cellular Sport Medicine, Institute for Cardiovascular Research and Sport Medicine, German Sport University Cologne, Cologne, Germany; 3 Heinrich Pette Institute, Leibniz Institute for Experimental Virology, Hamburg, Germany

**Keywords:** Endurance training, Marathon, HIV-infection

## Abstract

**Background:**

Improved treatment options of HIV have resulted in regular physical activities of many HIV-infected patients. However, data on effects of sports in HIV-patients are scarce.

**Methods:**

21 HIV-infected persons were monitored prospectively while preparing for a marathon run. Multiple parameters with regard to immunology, quality of life and metabolism were measured at 4 time points (at baseline 1 year before the marathon run, 3 and 6 months after beginning of training, and immediately before marathon).

**Results:**

13 out of 21 participants completed the marathon (12 male, 1 female; median age 42 years [27–50]; CD4 = 620/μl [146–1268]; 11 were on ART since 3.5 years [1–7]). 8 participants ceased training early. All reasons for stopping (besides one pre-existing metatarsal fracture) were not regarded as training-related (e.g. time limitation *n* = 3; newly diagnosed anal cancer *n* = 1; personal reasons/unknown *n* = 3).

We observed a significant increase in absolute CD4-T-cells (620/μl [146–1268] vs. 745 [207–1647]; *p* = 0.001) with simultaneous decrease of CD4-T-cell apoptosis (53% [47–64] vs. 32% [14–42]); *p* < 0.01). No effects on viral load independent of ART occurred. Systolic blood pressure and cholesterol improved significantly, although moderate and normal at baseline (cholesterol 185 mg/dl [98–250] vs. 167 [106–222], *p* = 0.02; RRsys 125 mmHg [100–145] vs. 120 [100–140], *p* = 0.01). Blood count, liver enzymes, creatinine and CK remained unchanged.

**Conclusions:**

The results of this pilot study indicated improved metabolic and immunologic parameters in HIV-infected patients undergoing moderate endurance training. Although training effects or ART cannot be ultimately separated as underlying mechanisms, we conclude that marathon training is safe for HIV-infected patients and potentially improves general health.

**Trial registration:**

DRKS00011592 (retrospectively registered on February 9th 2017).

## Background

The face of HIV infection has changed dramatically over the past decade. Due to continuous improvements in ART life expectancy approaches to that of the general population in most developed countries [1]. Quality of life has also improved for most treated patients, who are able to participate in daily activities without restrictions. Amongst other activities, strenuous exercise is undertaken by an increasing proportion of patients. However, HIV research has focused mainly on ART and associated problems such as efficacy, safety and tolerability, long term toxicities [2] and since recently prophylaxis [3]. Little is known about effects of usual activities such as exercise on HIV-associated clinical parameters and health state.

Effects of physical exercise on health have been discussed for decades. Most studies performed have focussed on cardiovascular disease. Impact of physical activities has been studied in other chronic diseases such as chronic obstructive pulmonary disease, chronic kidney disease, asthma and osteoporosis [4], diabetes type 2 [5], colon cancer [6] and dementia [7]. Studies in HIV-infected patients were conducted in the pre-ART era [8, 9] or addressed typical complications of HIV-infected patients such as wasting [10], lipodystrophy [11, 12], anxiety, depression or HIV-associated neurocognitive disorder (HAND) [13, 14]. However, studies on effects of physical exercise beyond the treatment of HIV-associated complications are scarce.

This is of particular interest as there is increasing evidence that immunologic functions and lymphocyte responses and proliferation are affected by exercise [15, 16]. It is the hypothesis that acute effects may be rather immunosuppressive whereas in the long-term fewer infections and better immune function are observed. Immunosuppression following exhaustive, acute activities might be due to numeric changes in peripheral white blood cells [17, 18] and functional changes such as reduced neutrophil function (chemotaxis, polarization) [19], IgA-secretion [20] and NK-cell activity [21]. Accordingly, the risk of upper respiratory tract infections (URTI) is increased in athletes by up to 2–6 times compared to the general population [22, 23], especially following a long-term period of heavy training and 1–2 weeks after a competition [22, 23]. In contrast, moderate exercise has a favourable long-term effect on the overall incidence of self-reported URTI. Studies showed up to 43% annual risk reduction [24] as well as a positive effect on severity and duration of URTIs [24]. Hence, the interplay between physical activities and immune function is of special interest in the HIV-infected population.

In the present study, we assessed the effects of moderate endurance training on HIV-infected persons. We analysed immunological and metabolic data of HIV-infected subjects performing regular moderate aerobic exercise over a period of one year their aim of running a marathon.

## Methods

### Participants

A total of 21 HIV-infected subjects starting moderate endurance training in order to participate in a marathon run (VIII Gay Games in Cologne, Germany) were followed prospectively for one year. Participants could apply nationwide via an internet-based procedure. Volunteers had to be at least 18 years and were required to have no restriction for running (e.g. heart insufficiency) certified by their treating physician. There was no restriction with respect to HIV-infection, i.e. AIDS present or not, on ART or not. Selection process took place centrally in a randomized and anonymous manner.

Study participants were provided an intensive program: individual training plans were established by supervising exercise scientists (iQathletik GmbH, Frankfurt, Germany). Psychological support, information on equipment and nutritional facts were provided.

Participants were monitored within the study. There was no influence on medical decisions of the treating HIV-specialists. Safety parameters were revealed after every visit and communicated to the patient only in case of immediate need for reaction (this was not the case).

Persons for a reference group were chosen as age-matched HIV-positive volunteers from the infectious diseases clinic of the university clinic of Bonn, Germany.

### Training

After pre training examination, participants trained three to four times per week following the individual training plans provided. Duration of training was about 3 to 4 h per week in the first training period.

Participants trained at 60–70% of maximum heart rate. Support was given directly by the exercise scientists. In the first twelve weeks the subjects’ endurance was gradually built up. Main goal of this training was to improve aerobic metabolism capacity.

After post training examination, weekly training hours were increased up to 6 h per week with the aim of economization of metabolism and cardiovascular system. Intensity of training was increased up to 70–80% of maximum heart rate.

Seven months after pre training examination, training duration increased up to 7 to 10 h per week of moderate endurance runs. For improvements of threshold performance and enzyme systems of anaerobic glycolysis, the participants had to also practice extensive sprints (e.g. 3*2 km with 800 m relaxed pace in between).

2 weeks before the marathon, participants trained only basic endurance at 60–70% of maximal heart rate.

### Parameters assessed

All Participants were monitored at central meetings, which took place at 4 time points – at 12 months before the marathon run, 9 months, 6 months and right before the run. At each time point following parameters were assessed: systolic and diastolic blood pressure, pulse, weight, CD4-T-cell count and viral load, fasting lipids and glucose, liver transaminases, creatine kinase, phosphate, differential blood count. In addition, CD4T-cell apoptosis was measured. Venous blood samples were taken in the early morning hours after overnight fasting before physical activity on the respective day. Participants reported adverse events such as infections via a self-reporting diary. They were observed for infectious complications four weeks after finishing the marathon.

Routine laboratory parameters were assessed in the central clinical laboratory of the University of Bonn.

Assessment of absolute numbers of CD4+, CD8+ T cells, B cells and NK cells was conducted by multi-parameter flow cytometry using BD Trucount tubes (Becton Dickinson, Heidelberg, Germany). Briefly, 50 μl of fresh whole blood was stained with BD Multitests comprising the following fluorochrome-labelled monoclonal antibodies (mAb); Panel 1: CD3-fluoresceinisothiocyanate (FITC), CD4-Allophycocyanin (APC), CD8-phycoerythrin (PE), CD45 peridinin chlorophyll protein (PerCP); Panel 2: CD3-FITC, CD16/CD56-PE, CD45-PerCP, CD19-APC. Following red blood cell lysis and fixation, cells (FACS lysing solution, Becton Dickinson) were analysed on a FACSCalibur and absolute cell numbers were calculated by BD MultiSet software (Becton Dickinson).

Levels of CD4-T-cells apoptosis were determined by Annexin V assay (Becton Dickinson). 100 μl of fresh whole blood was stained with CD3-PerCP and CD4-APC, washed and subsequently treated with BD Pharm Lysis buffer to remove red blood cells. After additional washing cells were stained with FITC-labelled annexin V and propidium iodide according to the manufacturer’s instructions. Cells were acquired on a FACSCalibur within 30 min post staining and subsequently analysed using FlowJo software.

Quality of life was assessed using the Medical Outcomes Study HIV Health Survey (MOS-HIV). The MOS-HIV is composed of 35 items investigating 11 dimensions and recalls the last 4 weeks. These are described in detail in [25]. Questionnaires were handed to the participants in the German translation of the MOS-HIV (German MOS-HIV Questionnaire, Version 2.1). It was provided by MAPI Research Trust, Lyon, France (MOS-HIV contact information and permission to use: MAPI Research Trust, Lyon, France. E-mail: contact@mapi-trust.org). Scaling and Scoring was performed according to the instructions provided.

### Statistical analysis

For statistical analysis GraphPad Prism (Version 3.0.3, GraphPad Software, La Jolla, USA) was used. Standard methods of descriptive statistics were applied (Student t-test for paired samples).

For calculation of the Homeostasis Model Assessment (HOMA) index the HOMA2 Calculator v2.2 (Diabetes Trials Unit, University of Oxford, http://www.dtu.ox.ac.uk/homacalculator/).

### Availability of data and materials

The datasets used and analysed during the current study available from the corresponding author on reasonable request.

The project was initiated, organized and sponsored by Abbott Pharmaceuticals (Wiesbaden, Germany; now Abbvie). However, the sponsor had no influence on scientific design and no access to results until completion of the project.

The study was approved by the Ethics committee of the University of Bonn (sequential number 138/09), which is in accordance with the provisions of the Declaration of Helsinki. Informed consent was given by all participants.

## Results

### Participant characteristics

**Fig. 1:**
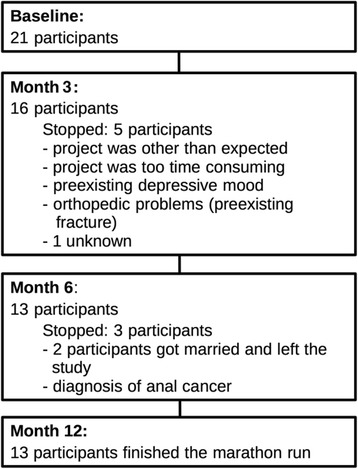
Characteristics of participants and reasons for training interruption

21 HIV-positive persons were enrolled to participate in the project. 8 participants did not finish the training period. Characteristics and reasons for training interruption are summarized in Fig. 1. Besides orthopedic problems in one person, all other reasons were regarded not to be related to the training.

The remaining 13 participants competed in the marathon run. All following data refer to these 13 participants with complete follow-up available.

### Baseline characteristics

Baseline characteristics of the 13 participants before training initiation are summarized in Table 1 (HIV-characteristics) and Table 2 (vital parameters and laboratory results).

**Table 1:** Baseline HIV-associated characteristics of 13 HIV-positive participants and 12 HIV-positive persons of the reference group

Parameter	Participants (*n* = 13)	Reference group (*n* = 12)
Sex	Male = 12 Female = 1	Male = 11 Female = 1
Age (years)	42 [27–50]	46.5 [26–57]
CD4 (/μl)	620 [146–1268]	571 [230–955]
CD4-Nadir (/μl)	230 [23–732]	315 [120–612]
AIDS diagnosis in history?	In 3 patients	In 1 patient
On ART?	Yes = 11 (6 NNRTI-based, 5 PI-based)No = 2	Yes = 6 (2 NNRTI-based, 4 PI-based)No = 6
In those on ART: Duration of ART (years)	3.5 [1–7]	4 [2–7]
HIV-RNA < 40 cop/ml	9/11One participant had a blip at baselineOne participant most likely had compliance problems	6/6
Comorbidity	2 chronic hepatitis 2 depression 1 psoriasis (dermal; no arthritis)	1 chronic hepatitis 2 depression

**Table 2:** Baseline vital parameters and laboratory results of 13 HIV-positive participants and 12 HIV-positive persons of the Reference group

Parameter	Participants (Baseline)	Reference Group	*p* (Participants vs. Reference group)
CD4-count (/μl)	620 [146–1268]	571 [230–955]	0.81
Annexin-binding (% of CD4+ cells)	53 [47–64]	51 [46–52]	0.12
Sodium (mmol/l)	139 [136–142]	139 [132–141]	0.58
Phosphate (mmol/l)	1.06 [0.86–1.20]	0.91 [0.66–1.19]	0.03
Weight (kg)	78 [58–100]	77 [59–88]	0.44
Height (cm)	174 [158–182]	179 [168–185]	
RRsys (mmHg)	125 [100–145]	115 [100–130]	0.06
RRdia (mmHg)	80 [70–90]	70 [60–90]	0.08
Leukocytes (G/l)	6.1 [4.1–9.5]	6.3 [3.8–10.6]	0.80
Hemoglobin (g/dl)	15.9 [11.3–17.3]	14.6 [12.9–16.2]	0.38
Thrombocytes (G/l)	225 [171–294]	255 [147–342]	0.50
INR	1 [0.9–1.2]	1 [0.9–1.1]	0.06
Potassium (mmol/l)	3.9 [3.6–4.5]	4.3 [4.0–4.9]	0.001
Creatinine (mg/dl)	1.05 [0.8–1.17]	0.96 [0.74–1.16]	0.31
Triglycerides (mg/dl)	156 [85–328]	141 [67–326]	0.95
Cholesterol (mg/dl)	185 [98–250]	177 [111–302]	0.98
HDL-cholesterol (mg/dl)	48 [31–60]	40 [28–54]	0.28
LDL-cholesterol (mg/dl)	115 [45–161]	111 [61–198]	0.81
Insulin (mIU/l)	4.4 [2.1–9.5]	6 [2–35]	0.15
Glucose (mg/dl)	86 [78–107]	91 [83–100]	0.17
Bilirubin (mg/dl)	0.56 [0.28–2.90]	0.47 [0.26–2.90]	0.70
ALT (U/l)	25 [10–126]	23 [10–53]	0.42
Creatine kinase (U/l)	105 [66–145]	86 [55–155]	0.64

**Table 3:** Changes in clinical and laboratory parameters throughout the training period

Parameter	Baseline	Month 3	Month 6	Month 12	*p* (Baseline vs. Month 12)
CD4-count (/μl)	620 [146–1268]	649 [198–1429]	791 [192–1419]	745 [207–1647]	0.001
Annexin-binding (% of CD4+ cells)	53 [47–64]	50 [37–60]	48 [25–57]	32 [14–42]	0.001
HIV-RNA (below <40 cop/ml)	10/11	10/11	10/11	10/11	
Sodium (mmol/l)	139 [136–142]	139 [137–141]	139 [136–142]	137 [135–139]	0.003
Phosphate (mmol/l)	1.06 [0.86–1.20]	1.13 [0.67–1.37]	1.12 [0.87–1.53]	1.19 [0.99–1.44]	0.02
Weight (kg)	78 [58–100]	77 [58–99]	78 [58–97]	77 [58–91.9]	0.01
RRsys (mmHg)	125 [100–145]	110 [110–140]	120 [110–140]	120 [100–140]	0.02
RRdia (mmHg)	80 [70–90]	80 [70–90]	80 [60–90]	80 [60–90]	0.04
Leukocytes (G/l)	6.1 [4.1–9.5]	5.0 [3.3–8.8]	5.7 [3.9–13.3]	5.5 [4.3–8.5]	0.37
Hemoglobin (g/dl)	15.9 [11.3–17.3]	15.4 [10.2–16.8]	15.6 [9.7–17.5]	14.8 [12.4–17.2]	0.15
Thrombocytes (G/l)	225 [171–294]	224 [164–325]	222 [134–338]	208 [160–316]	0.13
INR	1 [0.9–1.2]	1 [0.9–1.1]	1 [0.9–1.2]	1 [1–1.2]	0.27
Potassium (mmol/l)	3.9 [3.6–4.5]	4.4 [4.1–5.2]	4.0 [3.5–4.8]	3.9 [3.6–4.5]	0.31
Creatinine (mg/dl)	1.05 [0.8–1.17]	0.95 [0.66–1.17]	1.06 [0.68–1.23]	1.03 [0.79–1.16]	0.47
Triglycerides (mg/dl)	156 [85–328]	111 [84–224]	167 [83–306]	140 [82–262]	0.52
Cholesterol (mg/dl)	185 [98–250]	174 [131–227]	179 [117–214]	167 [106–222]	0.02
HDL-cholesterol (mg/dl)	48 [31–60]	42 [30–63]	39 [24–65]	39 [30–66]	0.41
LDL-cholesterol (mg/dl)	115 [45–161]	111 [73–160]	114 [61–154]	111 [55–141]	0.34
Insulin (mIU/l)	4.4 [2.1–9.5]	3.8 [2–15.7]	5.4 [2–16.8]	10 [2–18.8]	0.006
Glucose (mg/dl)	86 [78–107]	90 [79–109]	88 [62–114]	89 [70–109]	0.88
Bilirubin (mg/dl)	0.56 [0.28–2.90]	0.44 [0.24–2.94]	0.40 [0.19–2.23]	0.35 [0.20–1.63]	0.10
ALT (U/l)	25 [10–126]	25 [10–70]	23 [7–57]	21 [10–40]	0.11
Creatine kinase (U/l)	105 [66–145]	117 [84–192]	116 [50–240]	96 [62–226]	0.90

To convert to SI-units: bilirubin mg/dl × 17.104 = μmol/l; cholesterol mg/dl × 0.0259 = mmol/l; creatinine mg/dl × 88.4 = μmol/l; glucose mg/dl × 0.05551 = mmol/l; hemoglobin g/dl × 0.6206 = mmol/l; insulin mIU/l × 7.2141 pmol/l; phosphate mmol/l × 3.0974 = mg/dl; triglycerides mg/dl × 0.0114 = mmol/l

**Table 4:** Changes in parameters of the MOS-HIV

Parameter	Baseline	Month 3	Month 6	Month 12	*p* (Baseline vs. Month 12)
Physical Health Summary Score	58 [44–62]	59 [32–62]	59 [38–63]	56 [21–73]	0.26
Mental Health Summary Score	53 [38–61]	55 [35–66]	57 [29–64]	56 [23–68]	0.99
General Health Perception	70 [15–100]	70 [15–100]	80 [15–100]	75 [15–100]	0.94
Physical Functioning	100 [83–100]	100 [83–100]	100 [58–100]	100 [17–100]	0.21

For calculation and interpretation of scores please refer to [25].

The following parameters were in the normal range at baseline: leukocytes, hemoglobin, thrombocytes, international-normalized-ratio (INR), sodium, potassium, phosphate, creatinine, triglycerides, cholesterol, HDL-cholesterol, LDL-cholesterol, glucose, insulin, bilirubin, ALT, creatine kinase. No abnormalities were found in the urine dipstick analysis. Blood pressure was normal in all subjects (125 over 80). One patient was treated with an ACE-inhibitor due to moderate hypertension that was controlled under treatment. 11 participants were on ART while training period. 6 took a NNRTI-based therapy and 5 a PI-based regimen.

### Reference group

Laboratory parameters and vital parameters of the Reference group were assessed only at baseline to confirm, that the training group represents a representative sample of HIV-infected persons (Table 1). Longitudinal data were only assessed in the training group. At baseline, the following parameters showed no pathologic abnormalities: leukocytes, hemoglobin, thrombocytes, international-normalized-ratio (INR), sodium, potassium, phosphate, creatinine, triglycerides, cholesterol, HDL-cholesterol, LDL-cholesterol, glucose, insulin, bilirubin, ALT, creatine kinase. The average blood pressure was 115 over 70. One patient, suffering from hypertension, was treated with bisoprolol. As expected, there were no significant differences of the participants to the Reference group beside phosphate and potassium, which were in the normal range (Table 2).

6 persons of the Reference group did not take ART during the observation period. 4 were treatment naive, as they did not fulfill criteria for ART-initiation according to former treatment guidelines. However, all 4 started treatment in the longer follow-up. 1 patient had stopped ART due to toxicity and resumed ART 5 months after the study. 1 patient was regarded long-term non-progressor. He was lost to follow up thereafter.

### Effects of training

In general, training in the finisher group was tolerated without major complications. During the first few months during the winter period there were few infections of the upper airways. One patient was treated for kidney stones in month 10 of the training. However, he could resume the training and finish the marathon run. This patient was on atazanavir. No other adverse events were observed during the exercise period.

Investigating the potential effects of endurance training on key parameters of HIV infection we observed a significant increase in CD4-T cell counts following training initiation, while viral loads remained undetectable in fully suppressed individuals (Table 3). Interestingly, increasing CD4-T cell numbers were associated with a decline in the frequency of Annexin V+ CD4-T-cells, indicating reduced apoptosis-associated cell death as an underlying mechanism for the improved CD4-T-cell numbers.

A significant decrease in fasting cholesterol as well as systolic and diastolic blood pressure was observed. However, it has to be emphasized that all parameters were already in the normal range at baseline.

Overall, no significant change in quality of life was observed. All scales analyzed of the MOS-HIV showed no significant change (Table 4).

There were no differences in the other scores provided by the MOS-HIV: pain, role functioning, social functioning, energy/fatigue, mental health, health distress, cognitive functioning, quality of life, health transition (data not shown).

## Discussion

Regular physical activity is beneficial for HIV-infected patients, as for any human being. The intensity, frequency, and parameters of exercise for the population with HIV/AIDS have yet to be determined.

The main finding of our study was that regular aerobic activity resulting in performing a marathon was safe for people living with HIV. We could not show any potential harm, or increase of infections such as URTIs. Withdrawals from the study did not occur due to major health issues. There were no unfavorable effects on immunological and virological parameters.

In contrast we observed a significant increase in CD4 cell count by 20% (*p* = 0.001). Comparison to other studies is limited due to a vast variety of training schedules, duration and intensity. There might be an effect of the intensity of exercise: a meta-analysis of 14 studies in HIV-infected patients from 1990 to 2008 showed no difference in the CD4 cell count in the constant aerobic exercise group as well as in the moderate exercise group [26]. But there was a significant trend towards increase in CD4 cell count in the interval aerobic group, consisting of the data of two studies [9, 13].

Besides intensity another explanation of the differing results may be the compliance to training schedules, which is directly related to training frequency. This issue was further investigated by a study with evaluation of the compliance during training period [9]. A significant increase in CD4 cell count was shown in the compliant exercise group only while in the non-compliant exercise group a decrease was found. A more recent study on 10 participants with a combined training (resistance exercises plus aerobic training) over a period of 20 weeks showed an increase in CD4 cell count by 31%, but limitation of this study was the lack of a control group [27]. It is extremely difficult to adequately measure training-intensity of the participants. We tried to assess training intensity via self-reported diaries and log-files of GPS-watches in this study. However, data were not complete (mostly for technical reasons such as not activating log-files) and accurate enough to perform further detailed analyses.

The increase in CD4 counts in our study may be an effect of the longer training period compared to most studies also. We did not see a significant change in CD4 cell count after 3 months, but after 6 and 12 months. The training period of our project over one year is one of the longest being published so far. Most of the studies performed supervised exercise over a period of 12 weeks (7 of 10 studies in a recent meta-analysis [26]). Studies beyond this training period lasted for up to 24 weeks [8, 28, 29]. However, the studies with longest observation periods published by [8, 29] showed no effect on CD4 cell count. Studies on the immunological effects of aerobic training on the CD4/CD8 ratio in HIV-negative people showed conflicting results as well. Several studies observed no change [30, 31], while others found a positive alteration of CD4/CD8 ratio [32].

Interestingly, we observed a decrease of Annexin-V-binding lymphocytes in parallel to the increase in CD4 counts. A possible interplay between apoptosis and CD4+ cells was observed in numerous studies in non-HIV-infected athletes. During strenuous activities there may be changes in numbers of CD4+ cells (e.g. decrease of mainly Th1 cells but not Th2 cells) [15, 33], proliferation (decreased lymphocyte proliferation during and after exercise), probably due to an increase in apoptotic cells [34], and function of T-cells [35].

In our study the percentage of apoptotic cells was decreased after this long-term exercise. It is well-known, that following acute bouts of exhaustive exercise (> 75% VO_2_max) numbers of lymphocytes decrease and apoptotic cells increase [36–38]. However, long-term effects on apoptosis have not been reported. In addition, it remains unclear whether apoptosis is the main cause of lymphocyte dynamics related to exercise. It appears to be too small in number to be completely responsible for lymphocytopenia after exercise [18, 39, 40].

Going further, the effect of ART has to be considered. Some of the participants were on ART for just one year, therefore improvement of immunological status during the study period of one year is reasonable. Interestingly, few of the first studies on the effect of exercise in HIV-infection were performed before introduction of effective ART. Some studies showed an increase in CD4 cell count [41], whereas others did not [42, 43].

In summary, our data support a beneficial effect on the CD4 cell count, but study design and number of participants do not allow quantifying the direct effect of exercise on the lymphocytes and the underlying mechanism.

In general, moderate aerobic exercise has beneficial effects on metabolic parameters such as lipid profile and insulin. We found total cholesterol reduced at the end of the training period. It has to be emphasized, that cholesterol levels were already in the normal range in most patients at the beginning of the training period. Therefore, further considerations on effects have to be interpreted with caution. Nevertheless, positive results are in line with several studies on blood pressure, body fat mass and lipid profile of people living with HIV/AIDS [44, 45], where several beneficial effects such as an increase in HDL [27], a significant reduction in total adipose tissue (TAT), in cholesterol and triglycerides and an increase in HDL could be documented [46].

Noteworthy we found a significant increase in insulin-levels, while glucose levels were not changed significantly. Homeostasis Model Assessment of insulin resistance (HOMA-IR) index, calculated according to [47] increased within the training period from normal range to approximately >2 in some patients. This is surprising since moderate exercise has a beneficial effect on insulin resistance and glucose homeostasis control in diabetes patients [48] and HIV patients also [46]. The explanation for this finding remains unclear: Nine of those who finished marathon showed increasing insulinemia during the study period. Yet, none of the participants in our study had a history of diabetes or developed diabetes. 5 patients were on a PI-based regimen, and 3 of these patients had the greatest increase in insulin resistance of all patients on ART. The average increase in HOMA-index was two times higher compared to the patients on a NNRTI-based regimen. PI-based regimen have been shown to increase the risk of hyperglycemia by fivefold [49]. Two participants with decreasing dynamic of the HOMA-index (one over the complete study period) took a nevirapine containing regimen, that might have a favourable effect on insulin resistance [50]. One patient on a NNRTI based regimen remained stable with insulin resistance. The two HIV-positive patients without ART showed the greatest increase in insulin resistance.

Another finding was a significant increase in phosphate concentration. Tenofovir as part of many ART-combinations enhances phosphate excretion and has been associated with reduced serum phosphate levels [51, 52], but hypophosphatemia has a multifactorial genesis in HIV-positive patients and several analysis did not find a significant effect of tenofovir on the phosphate level [53, 54]. An increase of phosphate levels might indicate a partial reversal of this phenomenon. On the other hand, it might reflect increased cell turn-over due to exercise. It is not possible to conclude on any clinical significant effect.

Nevertheless, all changes in biological and metabolic parameters observed show – if relevant at all - an assumably positive effect.

We found no effect on the quality of life or mental health tests as assessed by MOS-HIV. Beneficial effects of exercise on these parameters have been described widely, but we have to assume that our quite small number of participants started training in a stable state of HIV-disease and state of quality of life. Possibly due to this fact, we could not document any relevant effect. However, we did not see any worsening of life quality of life in the MOS-HIV.

Limitations of our study are the lack of complete acquisition of cardiovascular parameters and training intensity, small number of participants, lack of control group, high drop out rate and difficulties in assessing outcome parameters as infections. It has to be emphasized that all HIV-infected participants were non-professionals, who lived their normal lives and contributed to this study in leisure time. Therefore, assessment could take place only during four time points, when participants met. In between, no reliable documentation other than self-reporting was possible. This is known to be highly variable. Especially severity and frequency of infections may be subject to personal variations. The overall drop out rate in our study was high with 38%. However, in most cases reasons were not related to illness but rather lack of time, marriage or feeling contrary to expectations of the project. Medical reasons for dropping out included pre-existing depression, an undiagnosed, most likely pre-existing metatarsal fracture and diagnosis of anal cancer and therefore probably only one health issue related to HIV. Noteworthy, all withdrawals took place in the first 6 months. Such a high drop out rate is a constant problem. It was estimated at the range from 3% [29] to 76% [8]. Nearly half of studies reviewed in a meta-analysis lost one third of participants [26]. However, high drop out rates in other study populations, such as non-HIV populations, are not unusual, especially in long-term studies (e.g. [55, 56]). This does not reduce the results of the study. Before the study we had a substantial discussion about the study design and about a control group. As the study mainly was designed to rule out negative effects of exercise rather than to detect CD4 dynamics, we decided to perform the study without a control group for two main reasons. Firstly, we would have needed a control group with a sedentary behaviour, which remained stable over the study period. Experience shows that the members of such a control group change behavior also, as they are enrolled in a study. Therefore, the reliability of such a study would have been substantially limited. Secondly, studies comparing of small-scale effects on lymphocyte dynamics would have required a much greater number of participants to adequately power the study. However, studies on effects of exercise on CD4 cell count have been carried out in pre-ART-era with varying results. Therefore we only expected small effects, if any. In the era of ART we had to rule out effects of a particular ART between the groups, which would have been impossible in such a setting with small numbers of participants. As efforts and costs for realization of this pilot-project were enormous and exceeded common clinical settings by far, the project targeted at 20 participants. More participants would have made this kind of project very difficult.

## Conclusions

In summary, HIV-infected persons can perform moderate endurance training. No clear safety issues were found in this “healthy” study population. These data pushed the safety limit of exercise in a specific group of people living with HIV/AIDS to the distance of a marathon run. In this small pilot study an increase in CD4-T-cells was observed, although it is not possible to conclude, whether this is a direct effect of training or due to ART. In a long-term perspective effects of endurance training on typical HIV-associated risks such as cardiovascular diseases would be desirable.

## Abbreviations

ART: anti-retroviral therapy
HIV: human immunodeficiency virus
HOMA-IR: Homeostasis Model Assessment of insulin resistance
MOS-HIV: Medical Outcomes Study HIV Health Survey
NNRTI: non-nucleoside reverse transferase inhibitor
PI: protease inhibitor
URTI: upper respiratory tract infections
